# DPU-ALDOSKI dataset of Monitoring and Controlling distributed far distances energy consumed system based on Internet of Things

**DOI:** 10.1016/j.dib.2023.109455

**Published:** 2023-07-28

**Authors:** Mohammed A. M． Sadeeq, Subhi R.M. Zeebaree

**Affiliations:** a ITM Dept., Technical College of Administration, Duhok Polytechnic University, Duhok, Iraq; b Energy Eng. Dept., Technical College of Engineering, Duhok Polytechnic University, Duhok, Iraq

**Keywords:** Sustainable-energy, Microcontroller, MQTT, Power saving

## Abstract

This article presents the created dataset obtained from implementation of a proposed distributed energy management system based on the Internet of Things (IoT). The proposed approach was implemented and evaluated at two distinct institutions, namely Duhok and Shekhan, which are geographically distant from each other. The proposed system comprises two primary phases, namely Monitoring and Controlling, which are implemented using ESP32 microcontrollers. The power usage indicators, namely Voltage, current, and Frequency, have been subjected to meticulous monitoring and regulation. Three principal methodologies are employed for each institution, namely: comprehensive monitoring of all metrics for the entire institution, comprehensive monitoring of all metrics for the uncontrolled laboratory, and monitoring and regulation of all metrics for the controlled laboratory. The technology in question continuously collects data, leading to the creation of a substantial energy management database. The data collection process involved the utilization of server-side protocols, namely Message Query Telemetry Transport (MQTT) and Hypertext Transfer Protocol (HTTP).

**Specifications Table**

**Table:** 

Subject	Energy Engineering and Power Technology
Specific subject area	Monitoring and Controlling Energy Management System based on Distributed IoT
Type of data	Table
How the data were acquired	Data: acquired *via* distributed IoT sensors covered two far distances Institutions. HW: There are three nodes in each institution. So, there are 6 ESP32 microcontrollers, 10 PZEM-004T, 4 SSR, 2 DHT22, 6 ULN2003, 6 Power Supply, 6 TFT Liquid Display. SW: Arduino C language, PHP, MySQL DB. Protocols: HTTP, MQTT.
Data format	Raw
Description of data collection	Conditions: The data were collected under real time institutions energy consumption. Factors: Data collected depending on the (Voltage, Current, Power, Frequency, Energy, Power Factor, Temperature, Humidity, Devices, Date, Time)
Data source location	Institution: Duhok Polytechnic university City/Town/Region: Duhok Country: Iraq
Data accessibility	Repository name: Mendeley Data [3] Data identification number: doi:10.17632/75c8p9kkfd.1 Direct URL to data: https://data.mendeley.com/datasets/75c8p9kkfd/1
Related research article	Mohammed A. M.Sadeeq, Subhi R. M. Zeebaree, Design and Implementation of an Energy Management System based on Distributed IoT, Computers and Electrical Engineering. 109 (2023) 108775. 10.1016/j.compeleceng.2023.108775

## Value of the Data

• These data are useful because they are obtained in a real-time manner.
• Number of records is more than (3,000,000) till now, and will be increase with time as continues growth.
• The data are created periodically by a rate of (2 records/Min) which provides (Hourly, daily, Weekly, Monthly, and Yearly) comparisons.
• All standard energy metrics are included such as: Energy, Power, Voltage, Current, Power Factor, Frequency, Date, and Time.
• Researchers who making comparisons among the IoT devices for energy management of different campuses can benefit from these data.
• These can be used for managing the energy consumed *via* intelligent algorithms. Finally, this dataset can be reused for providing the predicted minimum consumed energy.

## 1 Objective

The main reasoning of generation of this dataset is to provide Monitoring and Controlling energy management system based on distributed IoT devices. This data article can add value to the related article *via* providing certainty to the obtained results of the original article. This article will support the statistical analysis for the regression and prediction phases of the obtained results by the original article. Hence, increasing the size (number of records) of the created dataset continuously will provide more reliability to the Monitoring and Controlling processes of the original article. Also, allows the researchers to enhance and modify the depended models as future steps in this field.

## 2 Data Description

The main metrics description of the created dataset which named (DPU-ALDOSKI) are illustrated in Table 1. There are fifteen metrics declared the collected data records of the created dataset. Some of these metrics need to be denoted with their (minimum and maximum) acceptable limits. So, acceptable ranges of the (Voltage, Frequency, and Power_factor) are (200-240V, 48-52Hz, and 0.5-0.9) respectively.

**Table 1:** Description of the used metrics for the DPU-ALDOSKI dataset.

#	Metric	Description
1	Voltage	Measured voltage (Volt)
2	Current	Measured current (Ampere)
3	Power	Measured power (Watt)
4	Frequency	Measured frequency (Hz)
5	Energy	Measured consumed energy (KW/Sec)
6	Power_Factor	Percentage ratio of the True Power/ Apparent Power
7	Temperature	Room Temperature C^o^
8	Humidity	Room Humidity %
9	Device No.	Microcontroller Board number
10	Split State	Status of the room air-conditioning device (ON/OFF)
11	Computers State	Status of the Lab computers and light (ON/OFF)
12	Date-Time	The timestamp of each record data
13	Date	The date of each record data
14	Time	The time of each record data
15	Date_Time	The date and time of each record data (in Varchar format)

The uploaded records of the dataset are from (13 Feb 2022) to (8 January 2023), there are (3,070,625) records available in it. But, as a policy of the depended DPU-ALDOSKI dataset, the recording process of the collected data is automatically and continuously done for the both institutes (Duhok and Shekhan) since (13 Feb 2022) and ongoing. Consequently, the number of collected records for this dataset will be increased sharply with the time. This large dataset provides more reliability to the researchers who want to use this dataset any time he/she wants [1].

The proposed EM system comprises two fundamental constituents, namely Nodes and the Main Control Unit (MCU). Every individual Node represents a campus, and within each campus, there exist three microcontroller devices functioning as sub-nodes. Three discrete designs will be created for each Node, contingent upon the sub-nodes. In contrast, the MCU pertains to cloud computing, specifically Blynk-Cloud or web hosting. The system under consideration comprises of two distinct nodes, and can be extended to N nodes in future. In order to collect information, every Node is equipped with a set of sensors. The information provided will be utilized by the system to make sound judgements [1,2].

## 3 Experimental Design, Materials and Methods

The proposed system relies on three primary architectures for its design and implementation: Institution Load Measurement Architecture (ILMA), Uncontrolled Load Measurement Architecture (ULMA), and Full Controlling Load Measurement Architecture (FLMA). Various architectural designs have been suggested based on specific duties and primary objectives [1].

### 3.1 Institution Load Measurement Architecture (ILMA)

The (Institution) architecture is accountable for quantifying the aggregate values of the reliant metrics associated with the entire edifice of the campus being tested. The academic entity in question can be classified as a University, College, or Institute. The measured data is transmitted at regular intervals of 30 second. The MCU proceeds to store all received data for subsequent analysis and extraction of pertinent reports in the form of crucial statistics.

### 3.2 Uncontrolled Load Measurement Architecture (ULMA)

The architectural design in question is tasked with the measurement of aggregate electric metrics pertaining to a specific room or laboratory located within the Unit campus undergoing testing. Furthermore, this architectural design has the capability to oversee all of the incorporated devices within the unit being tested.

### 3.3 Full Controlling Load Measurement Architecture (FLMA)

The architectural design in question is tasked with the measurement of aggregate values of metrics that are dependent on a particular Room or Laboratory within the Unit, which is the campus currently undergoing testing. Furthermore, this particular architectural design has the capability to oversee and regulate all of the incorporated devices within the unit being tested.

## Ethics Statements

The Duhok Polytechnic University and both its institutes (Duhok and Shekhan) approved the study. The research was carried out in accordance with the mandatory needs of power consumption reduction in Iraq and Kurdistan Region.

## CRediT authorship contribution statement

**Mohammed A. M．  Sadeeq:** Data curation, Writing – original draft, Formal analysis, Visualization, Investigation, Methodology, Software, Writing – review & editing. **Subhi R.M. Zeebaree:** Supervision, Conceptualization, Methodology, Writing – review & editing.

## Data Availability

DPU-ALDOSKI (Original data) (Mendeley Data).
